# Development of Temperature Control Solutions for Non-Instrumented Nucleic Acid Amplification Tests (NINAAT)

**DOI:** 10.3390/mi8060180

**Published:** 2017-06-07

**Authors:** Tamás Pardy, Toomas Rang, Indrek Tulp

**Affiliations:** 1 Thomas Johann Seebeck Department of Electronics, Tallinn University of Technology, Ehitajate Tee 5, 12616 Tallinn, Estonia; toomas.rang@ttu.ee; 2 Selfdiagnostics Deutschland GmbH, 04103 Leipzig, Germany; indrek.tulp@selfdiagnostics.com

**Keywords:** lab-on-a-chip, LoC, finite element modelling, resistive heating, Point-of-Care, PoC, temperature control, computer aided design, microfluidics, isothermal nucleic acid amplification tests, NINAAT, NAAT

## Abstract

Non-instrumented nucleic acid amplification tests (NINAAT) are a novel paradigm in portable molecular diagnostics. They offer the high detection accuracy characteristic of nucleic acid amplification tests (NAAT) in a self-contained device, without the need for any external instrumentation. These Point-of-Care tests typically employ a Lab-on-a-Chip for liquid handling functionality, and perform isothermal nucleic acid amplification protocols that require low power but high accuracy temperature control in a single well-defined temperature range. We propose temperature control solutions based on commercially available heating elements capable of meeting these challenges, as well as demonstrate the process by which such elements can be fitted to a NINAAT system. Self-regulated and thermostat-controlled resistive heating elements were evaluated through experimental characterization as well as thermal analysis using the finite element method (FEM). We demonstrate that the proposed solutions can support various NAAT protocols, as well as demonstrate an optimal solution for the loop-mediated isothermal amplification (LAMP) protocol. Furthermore, we present an Arduino-compatible open-source thermostat developed for NINAAT applications.

## 1. Introduction

Lab-on-a-Chip (LoC) non-instrumented nucleic acid amplification tests (NINAAT) are novel molecular diagnostics devices promising to bring high-accuracy, portable Point-of-Care rapid tests. Isothermal nucleic acid amplification protocols require a single well-defined temperature range for a specific incubation time, and are particularly well-suited for NINAAT applications [1,2]. However, the platform introduces constraints on physical size, power dissipation, and material costs, which pose a unique challenge for temperature control solutions. At present, off-the-shelf solutions for temperature control in LoC NINAAT devices are not readily available, which limits commercialization efforts.

Most reported LoC systems are instrumented and rely on external means for temperature control [3,4,5], which decreases their portability. A fully portable system requires an integrated solution. Most commonly, electrical heating elements are integrated, primarily Peltier cells [6,7]. Another popular solution is to use a thin film heating element [8,9,10], such as a resistive heater or a micro-Peltier cell. In the case of microfabricated LoC systems, the thin film heater can be integrated on the same substrate as the fluidic channels, resulting in a more compact and power-efficient layout. Integrated self-regulating resistive heating is a viable candidate for NINAAT applications [11], as it does not require the addition of a thermostat; however, its power efficiency is still an issue that limits portability. An interesting alternative is offered by chemical heating, which is a heating element based on a highly exothermic chemical reaction of two or more components [12,13], typically an oxidative process regulated by the addition of a phase-change material (PCM). Advantages are their low cost and simplicity; disadvantages are their large size and the difficult regulation of their temperature output.

Many LoC systems with integrated temperature control methods are designed for polymerase chain reaction (PCR) [9,14,15] rather than isothermal nucleic acid amplification test (NAAT) protocols [2,15]. PCR requires thermal cycling, that is, rapid incubation temperature changes, which is even more challenging to implement in a chip format than a single, well-defined incubation temperature.

Resistive electrical heaters are well-known and well-defined, and therefore pose the lowest development risk for an integrated microheating solution. Injection molded plastics as the substrate of choice for LoC are becoming popular [16], primarily due to the low cost and the high aspect ratio achievable in mass-production. However, mold design and manufacturing is expensive, and therefore errors in geometry design are costly to correct. Finite element thermal analysis can greatly reduce development time by providing a tool to analyze and thermally optimize device geometry. Most research efforts have targeted the thermal analysis of integrated heating elements developed in-house, primarily within the same clean room process as the fluidic chip itself [17,18,19]. However, injection molded plastics require a separately manufactured heating element—preferably a commercially available heating element—which requires optimization of the thermal system.

In previous works, we investigated finite element modelling as a tool for the thermal analysis of various commercially available heating elements [20,21,22]. In this work, we demonstrate the process by which commercially available resistive heating elements can be evaluated for use as temperature control solutions for NINAAT systems. We also demonstrate an optimal temperature control solution for isothermal NAATs in a LoC format. We evaluate a self-regulated and thermostat-controlled resistive heating solution through experimental characterization as well as thermal analysis. Furthermore, we present an Arduino-compatible open-source thermostat developed for NINAAT applications.

## 2. Materials and Methods

### 2.1. Thermal Analysis via Finite Element Modelling

For thermal analysis, we use a Joule heating model with terms added for the specific heating element used. The initial assumption is made that the liquid in the simulated microreactor cavity is stationary for the duration of heating. The model consists of two sets of equations. The heat generation (for a known and experimentally characterized heating element) is expressed by the formula of power $\frac{\mathrm{dP}}{\mathrm{dV}}=J\cdot E$ [23], where P [W] is the power, V [m^3^] the volume of the heating element, J [A/m^2^] the current density, and E [V/m] the electric field. The generated heat is propagated through the geometry of the model by the heat transfer equation [24]. Fields J and E are derived from the following set of equations [25]:

(1) $$\nabla J=Q_{j}\;J=\left(\sigma +\varepsilon_{0}\varepsilon_{r}\frac{\partial }{\partial t}\right)E+J_{e}\;E=-\nabla V$$

where $Q_{j}$ [$A/m^{3}$] are current sources (sinks), $J_{e}$ [$A/m^{2}$] the external current density (if there is one), V [V] the potential drop in all directions, σ [S/m] the conductivity, $\varepsilon_{0}$ the relative permittivity of vacuum, and $\varepsilon_{r}$ the relative permittivity of the material at the point of the geometry where the equations are evaluated. The materials in the simulated geometry are characterized electrically through their conductivity and relative permittivity, whereas heat transfer properties are characterized by their density ($\rho \;\left[\mathrm{kg}/m^{3}\right]$), heat conductivity (k [W/(m⋅K)]), and specific heat capacity ($C_{p}\;\left[J/\mathrm{kg}\cdot K\right]$) for each spatial point of the geometry. The heat losses to the ambient (i.e., via convection) are defined as boundary conditions on the appropriate boundaries. The assumption is made that the modelled device is in a closed, controlled environment, with restricted ambient temperature variations, and ventilation kept at a minimum for the observed duration. Self-regulating resistive heating elements contribute to the conductivity term in Equation (1) as the reciprocal of the temperature-dependent resistivity of the heater substrate. This can be represented in one of three ways:

(1) If the heating element is electrically and structurally well-defined, the resistivity profile is directly fed into the model.
(2) For a rough approximation of heating elements that are not well-defined, the linear approximation of resistivity [26] is used: $R\left(T\right)=R_{0}\cdot \left[1+\alpha \cdot \left(T-T_{0}\right)\right]$.
(3) For PTC thermistors with known coefficients, the Steinhart–Hart equation is used [27].

Thermostat-regulated heating elements contribute to the electric potential in Equation (1) by adding a variable potential on the input terminals of the heating element. For the demonstrated proportional control algorithm, the input was expressed as follows [28]:

(2) $$V_{\mathrm{in}}\left(t\right)=K_{p}\cdot e\left(t\right)$$

where $V_{\mathrm{in}}\;\left[V\right]$ is the potential drop on the heating element, $K_{p}\;\left[1/K\right]$ the proportional controller gain, and $e\left(t\right)=T_{\mathrm{set}}-T_{\mathrm{current}}$ the process error at time instant t [s]. $T_{\mathrm{set}}\;\left[K\right]$ is the temperature set point of the control algorithm, and $T_{\mathrm{current}}\;\left[K\right]$ is the measured temperature at time instant t.

The finite element model was implemented in COMSOL^®^ Multiphysics (version 5.2) using the Heat Transfer and Electric Currents interfaces coupled through the Joule Heating interface. All models were three-dimensional and the model geometry was imported from Autodesk Inventor. The boundary conditions and the initial values were derived from the physical prototypes used for experimental characterization. For the steady-state thermal analysis (Section 3.1), COMSOL’s Stationary solver was used, whereas for the spatiotemporal analysis (Section 3.2), the Time-Dependent solver was used. All the domains of the physical prototype device were taken into account in the simulation, but the geometry was defeatured to decrease mesh complexity. The wires, screws, and labels were removed. Where applicable, axes of symmetry were used to cut the geometry. The convective, conductive, and radiative heat losses were calculated on the external boundaries. The temperature sensors in the physical prototype were modelled by Domain Point Probes. The proportional control algorithm was implemented as a user-defined equation, and coupled to the input voltage boundary condition. For both models, COMSOL generated a tetrahedral mesh. The average mesh element quality—based on the widely used radius ratio method [29]—for the self-regulated model was 0.674 (element size: 0.21 mm^3^), whereas for the thermostat-regulated model it was 0.673 (element size: 0.18 mm^3^).

### 2.2. Open-Source Thermostat

An open-source mini-thermostat was developed for temperature regulation of isothermal NAAT protocols in LoC devices. The primary implementation was on an Arduino Uno demo board, with an additional circuit board developed for taking temperature sensor readouts and applying proportional control on the current input of a resistive heating element via a metal-oxide semiconductor field-effect transistor (MOSFET) switch. Proportional control was chosen over proportional-integral-derivative (PID) control for its minimal computational space requirements, and to ensure easy downscaling further on in the development process for integrated controller solutions, while maintaining enough space for the integrated timer and user feedback functionalities. The thermostat was packaged in an enclosure and equipped with a piezo buzzer for auditory feedback. A graphical user interface was implemented in the Microsoft .NET framework to control the thermostat from a PC through a serial connection. The primary use case for this prototype is to connect a two-wire thermistor probe for sensing and a two-wire resistive heating element for temperature control in a LoC NAAT system. The user can configure the desired assay time and target temperature, and the thermostat will beep to indicate when the reaction is done. The thermostat can be powered from a wall socket using a standard direct current (DC) transformer, or alkaline batteries with at least 5 V output (depending on the specifications of the heating element). Please refer to the Appendix A for more information and schematics.

### 2.3. Plastic NINAAT Thermal Models and Measurement Setup

**Figure 1:**
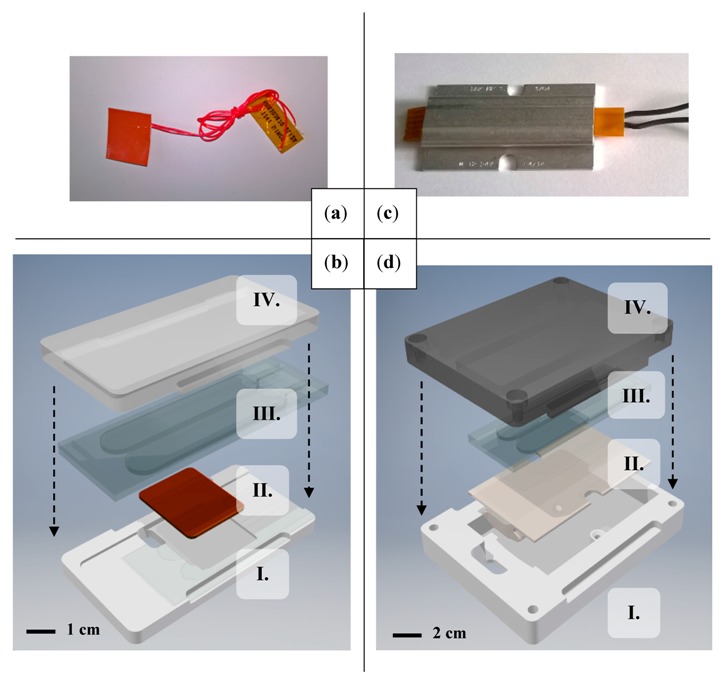
Minco HR5303 heating element for thermostat-regulated temperature control evaluations (**a**) DBK HP04 heating element for self-regulated temperature control evaluations; (**c**) the prototype for evaluating self-regulated heating; (**d**) consists of a milled plastic enclosure (I. and IV.), the plastic nucleic acid amplification test (NAAT) chip model (III.), and the heating element (II.). The prototype for evaluating thermostat-regulated heating (**b**) had the same basic layout and a different heating element.

For the experimental thermal characterization, two setups were created based on polymethyl methacrylate (PMMA) plastic prototypes manufactured by milling. The centerpiece was a model for a disposable microfluidic NAAT chip (25 mm × 75 mm area), with two reactor cavities designed to hold up to 0.7 mL of reaction volume each. Two enclosures were created for the two heating elements with slots for the chip (Figure 1b, d).

The NAAT mock-chip had two two-wire 10-kΩ negative temperature coefficient (NTC) thermistor probes sealed into each reaction chamber. One thermistor was connected to an Agilent 34410A digital multimeter for temperature recording. The other probe was only used in thermostat-regulated heating experiments as the temperature input for the thermostat. The heating elements were powered from an Agilent E3631A triple-channel DC power supply (Agilent Technologies Inc., Santa Clara, CA, USA). Two-digit precision was used for recording temperatures as well as the output current values of the power supply. The power supply was operated as a voltage source (output set with two-digit precision) and a 1 A current limit was applied.

### 2.4. Heating Elements and Thermal Interface

The self-regulating heating element discussed in this paper (Figure 1a) was a ceramic PTCR (positive temperature coefficient of resistance) heater produced by DBK (model DBK HP04-1/04-24, DBK David + Baader GmbH, Rülzheim, Germany). It consisted of a ceramic heating element and an aluminum profile with a flat surface, originally designed for enclosure heating. Its operating voltage range by design was 1–30 V_DC_ [30]. The PTCR effect entails a rapid increase in substrate resistivity with elevated temperatures, which limits input current on the heating element and thus its heat output. Although structural or material data was not disclosed by DBK, most ceramic PTCR heaters are constructed from doped polycrystalline barium titanate (BaTiO_3_) ceramics [31]. The onset of the PTCR effect occurs at the Curie temperature (T_c_) of the material that marks a transition from the ferroelectric to the paraelectric phase. Dopants such as strontium or lead [32] can shift T_c_ and thereby define temperature set points as needed by the application. Besides material properties, the input voltage affects the onset of the PTCR effect as well, enabling accurate temperature control in a well-insulated device, as will be demonstrated in this paper.

For thermostat-regulated heating, an etched foil heating element (Figure 1c) from Minco Products Inc. was used (P/N HR5303R70.2LI2A, Minco Products Inc., Fridley, MN, USA). These heaters typically rely on an etched resistive film (e.g., chrome–nickel) as the heater substrate, encased in polyimide and silicone rubber sheets. According to manufacturer data, the element used had a base resistance of 70.2 Ω [33].

Initially, the heating elements were characterized electrically and thermally. The resistance values were measured at room temperature using a digital multimeter (Agilent 34410A, Agilent Technologies Inc., Santa Clara, CA, USA). The temperature dependence of the self-regulated heating element was modelled by linear approximation (see Section 2.1). The heating element was encased in a polyurethane mold for thermal insulation, then connected to the power supply (Agilent E3631A), and the input current recorded at 1–24 V input voltage with 1 V steps while recording surface temperatures. At each step, the heater was allowed to reach a steady state and then to cool down to room temperature. The reference temperature was defined as 24 °C (room temperature), and monitored with a digital thermometer. The temperature coefficient of resistivity was calculated at 0.16 (±0.09) [1/K], whereas the base resistance at room temperature was measured at 10 (±0.4) Ω. 

The resistance of the etched foil heating element was considered constant in the temperature range studied, and was measured at 76.38 ± 0.37 Ω [20], rather than the 70.2 Ω [33] disclosed by the manufacturer. 

The thermal structure was similar in both experimental setups. The heating element was directly interfaced with the plastic NAAT chip, and the temperature sensors were encased in the microreactor cavities of the chip. No thermal interface material was used, as the system was designed for removable single-use NAAT chips.

## 3. Results and Discussion

### 3.1. Steady-State Performance for Multiple NAAT Protocols

**Figure 2:**
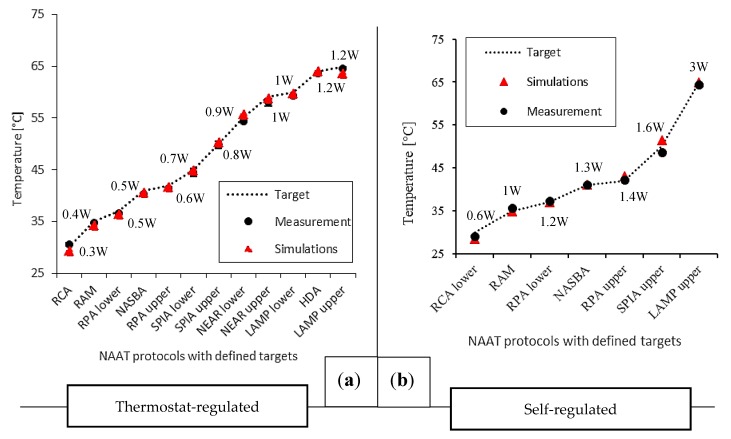
Steady-state thermal characterization and performance evaluation of commercial heating elements in test setups modelling non-instrumented nucleic acid amplification test (NINAAT) systems for multiple NAAT temperature targets ranging from 30 to 65 °C (**a**,**b**). A finite element thermal model was set up and trained for the heating element used. The model was demonstrated to accurately estimate steady-state temperature outputs, and is useable as a tool for spatial thermal analysis to evaluate temperature control performance.

As the first step in the optimization process of temperature control for NINAAT systems, an experimental thermal characterization was performed for both experimental setups. The thermostat-regulated setup was run with 15 V input voltage at a 1 A current limit and various temperature set points. The proportional gain of the thermostat was set to 20 [1/K] for rapid heating. The self-regulated setup was run with various voltage inputs in the 5–24 V range with a 1 A current limit. In both cases, target temperatures for the evaluation were selected from a set of targets for various NAAT protocols as listed in Table 1, and steady-state temperatures were recorded for evaluation. The ambient temperature was maintained at 22–24 °C and monitored with a digital thermometer. The experimental conditions were set according to the targeted application (Point-of-Care (POC) testing), thus the device prototype was tested in free air, in a closed laboratory environment with minimal ventilation. A climate chamber was not used for testing, as any prospective end-user device is intended for use in offices and at home, where some ventilation is present. The results are shown in Figure 2. For the self-regulated heating evaluation, the steady-state error (SSE) was 0.59 °C (±0.39 °C) for the selected temperature targets, whereas for the thermostat-regulated evaluation it was 0.53 °C (±0.19 °C). Considering the average of all experiments, the controller regulated temperatures had a ±1 °C accuracy around the set point, which is a suitable accuracy for most isothermal NAAT protocols.

The second step was to set up and train our finite element model for thermal analysis, given the two heating elements under study and the experimental setups. Both setups were modelled in COMSOL^®^ Multiphysics and the boundary conditions were defined according to experimental parameters. The heating elements were fully defined, including their internal structure. The material properties were either literature or COMSOL library values, except for the resistivity profiles of the heating elements. The total element number (tetrahedral domain elements) for the generated mesh was 195114 for the self-regulated setup and 338064 for the thermostat-regulated setup. Both setups had nine simulated domains, but in the self-regulated setup only half of the model was simulated due to the axial symmetry. The model was solved on a PC with a Core i5-4570 CPU and 16 GB RAM. The typical execution time for a single set of parameters ranged from 2 min to 55 min depending on complexity. Complexity was heavily affected by the rate of change in the simulated system, especially since the thermostat-regulated system included a feedback loop for the controller. For the self-regulated heating setup, the model estimated experimental results with a mean absolute error (MAE) of 0.96 °C over the defined set of input parameters. For the thermostat-regulated setup, MAE was 0.64 °C. A summary of the steady-state measurements and the simulation results are shown in Figure 2.

For the selected temperature targets, the control precision was similar in both systems; however, the thermostat-regulated system offered a higher degree of freedom in choosing the target temperature. The self-regulated setup was limited to a lower number of targets. Therefore, the thermostat-regulated system was proven a better candidate for this temperature control scenario.

### 3.2. Spatiotemporal Performance Analysis for Specific NAAT Protocol

Loop-mediated isothermal amplification (LAMP) is a highly sensitive and specific DNA amplification method developed by Eiken Chemical Co. [15], which requires incubation for 15–60 min at 60–65 °C temperature. We chose LAMP for this evaluation due to its high-temperature requirement that makes it very challenging to support in a portable, and especially disposable, system. We targeted at least 15 min in the prescribed temperature range with our temperature control solutions. First, we attempted to demonstrate with experimental thermal characterization that both prototypes could reach the target. Then, using the thermal models that we previously adjusted to the heating elements, we performed a thermal analysis to compare the performance of the two temperature control solutions. The setups were configured for the specified target range based on the input parameters defined in the previous section (Figure 2).

**Figure 3:**
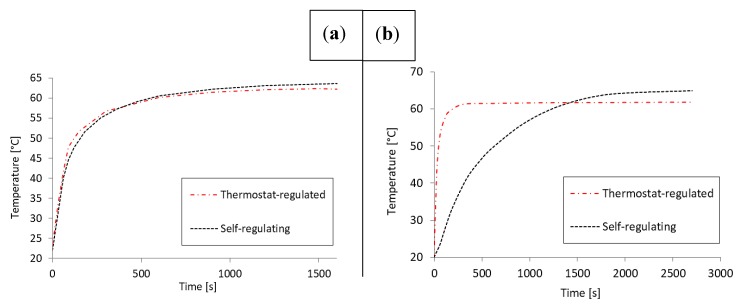
Temporal analysis specifically for the LAMP NAAT protocol that required 60–65 °C reaction temperature. Experimental characterization (**a**) indicated time constants below the required 15 min as well as successful regulation within the set temperature range for both solutions. The thermal transient simulation indicated a more pronounced difference: the setting time was significantly lower for the thermostat-regulated setup than the self-regulating setup (**b**).

The time constants were determined experimentally. The self-regulated setup reached the control target after 7.5 min during the experimental characterization, whereas the thermostat-regulated setup reached it in 9 min. The self-regulated setup reached steady-state in 35 min, and the thermostat-regulated setup reached it in 10 min. No overshoot was detected. Both temperature control solutions could maintain the target range for a minimum of 15 min. Figure 3 shows a comparison of results between the two setups.

The finite element models were solved as described previously for both setups using the same input parameters as during the experiments. The solution time was 20 min for the self-regulated setup, and up to 1.5 h for the thermostat-regulated setup due to the increased complexity resulting from higher rates of change. The self-regulated heating model estimated experimental results with an MAE of 9.15 °C, whereas the thermostat-regulated model had an MAE of 4.55 °C. However, in steady-state, the error decreased to 0.36 °C and 0.5 °C respectively. The measured time constants were estimated with an MAE of 8.7 min for the self-regulated setup and 5.5 min for the thermostat-regulated setup.

The higher error for the self-regulated heating model resulted in part from the linear resistivity model chosen for its simplicity. The linear model fails to capture the non-linearity of temperature-dependent resistivity inherent in ceramic semiconductors. Furthermore, it is not possible to accurately represent thermal interfaces and thermal capacities in a model due to the limited amount of data available on structural details, as well as the necessity to simplify the geometry representations in order to fit them into the available computational space.

**Figure 4:**
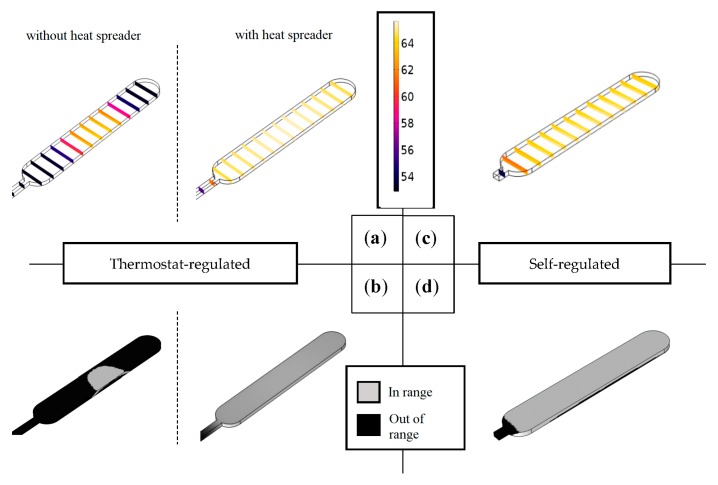
Application of finite element thermal analysis: steady-state temperatures are shown inside the reaction chamber for both the thermostat-regulated (**a**) and self-regulated experimental setups (**c**) visualizing heating efficiency. Visualizing the reaction volume in the target range (**b**,**d**) reveals deficiencies in heat transfer and insulation. The installation of a heat spreader improves the performance of the thermostat-regulated setup to the level of the self-regulated setup.

The model was applied in a spatiotemporal thermal analysis to determine the volumetric heating efficiency $\eta_{h}$ in the two test structures. The evaluation was performed based on the following expression:

(3) $$\eta_{h}=\frac{1}{\mathrm{coord}}\cdot \underset{\mathrm{coord}}{\sum }\left(T>59.99\left[{}^{\circ}C\right]\cap^{\text{​}}\;T<65.01\left[{}^{\circ}C\right]\right)$$

where coord denotes the spatial coordinates (x, y, z), and $\eta_{h}\in \left[0;1\right]$. First, this expression was evaluated in steady-state to determine the maximum heating efficiency for both setups for a direct comparison. In the self-regulated setup, 95% of the reaction volume was in range (0.66 mL out of 0.7 mL). In the thermostat-regulated setup, 8% of the reaction volume was in range (0.06 mL). However, adding a heat spreader—a 1 mm thick aluminum sheet—to the heater–chip interface increases the reaction volume in range to 95% (0.66 mL). The results are shown in Figure 4.

The experimental evaluation indicated that the self-regulated setup reached the control target 1.5 min earlier than the thermostat-regulated setup, whereas steady-state was reached 25 min earlier in the thermostat-regulated system. The controller gain was set high to ensure rapid heating, but the algorithm cut down the output near the set point to prevent overheating, which resulted in a longer heat up. The power input required by the self-regulated setup was 9 V higher than for the thermostat-regulated setup, making the latter more favorable for portable applications. However, the presented plastic prototypes were created to fit the dimensions of the heating elements rather than optimal power efficiency. The demonstrated setups could be powered using 2–3 pieces of 9 V alkaline batteries, which would form a major part of any portable device using this system. However, reducing the size of the heated area and the heating element in turn reduces the size of the power supply, and therefore the size of the whole device. In any NINAAT design scenario, the reaction chamber size must be minimized and heat transfer efficiency on the chip–heater interface maximized.

To summarise, at the present stage of development, the self-regulated setup offers better temperature control performance for the LAMP protocol (the incubation target temperature is reached faster). Additionally, despite the fact that it needs a more sizeable power supply than the alternative, it does not need the thermostat, which reduces cost and complexity. Including a battery compartment, this NINAAT temperature control solution would have dimensions of 8 cm × 6 cm × 4 cm. The thermostat alone has dimensions of 11 cm × 6 cm × 4 cm. Therefore, for an integrated test device, the overall footprint is smaller for the self-regulated system. This makes it more favorable for portable applications.

## 4. Conclusions

We demonstrated a process by which commercial off-the-shelf heating elements can be evaluated for and built into Lab-on-a-Chip devices that require precise temperature control. The primary application area for this technique is non-instrumented isothermal nucleic acid amplification in a chip format, where the heating element is a major technical challenge and a potential hurdle that slows down the commercialization of these devices. Two commercially available heating elements were evaluated and compared as potential temperature control solutions, and an optimal candidate was proposed. The demonstrated solution was capable of creating and maintaining the temperature range required for the loop-mediated isothermal amplification protocol inside a microreactor cavity within a time range expected from a rapid test. The proposed finite element model for thermal analysis can help optimize device geometry and insulation, as well as evaluate volumetric heating efficiency.

## Figures and Tables

**Table 1:** Target temperatures for isothermal nucleic acid amplification tests (NAAT) protocols [15].

Method	Full Name	Target	Steady-State Error (SSE) (Self-Regulated)	SSE (Thermostat)
NASBA	Nucleic Acid Sequence Based Amplification	41 °C	0.1 °C	0.6 °C
HDA	Helicase Dependent Amplification	64 °C	Not tested	0.3 °C
LAMP	Loop Mediated Isothermal Amplification	60–65 °C	0.6 °C	0.3 °C
NEAR	Nicking Enzyme Amplification Reaction	55–59 °C	Not tested	0.7 °C
RCA	Rolling Circle Amplification	30–65 °C	0.9 °C	0.6 °C
RPA	Recombinase Polymerase Amplification	37–42 °C	0.2 °C	0.2 °C
SPIA	Single Primer Isothermal Amplification	45–50 °C	1.2 °C	0.2 °C
RAM	Ramification Amplification Method	35 °C	0.75 °C	0.2 °C

## Appendix A

Hardware schematics and code for a basic implementation of the mini-thermostat are available at https://www.hackster.io/salhar1/mini-thermostat-e732ed, under the GPL3+ license.

## References

[1] Yager P., Edwards T., Fu E., Helton K., Nelson K., Tam M.R., Weigl B.H. (2006). Microfluidic diagnostic technologies for global public health. Nature.

[2] Chang C.-C., Chen C.-C., Wei S.-C., Lu H.-H., Liang Y.-H., Lin C.-W. (2012). Diagnostic Devices for Isothermal Nucleic Acid Amplification. Sensors.

[3] Rodriguez N.M., Wong W.S., Liu L., Dewar R., Klapperich C.M. (2016). A fully integrated paperfluidic molecular diagnostic chip for the extraction, amplification, and detection of nucleic acids from clinical samples. Lab Chip.

[4] Kim T.-H., Park J., Kim C.-J., Cho Y.-K. (2014). Fully Integrated Lab-on-a-Disc for Nucleic Acid Analysis of Food-Borne Pathogens. Anal. Chem..

[5] Mao H., Yang T., Cremer P.S. (2002). A Microfluidic Device with a Linear Temperature Gradient for Parallel and Combinatorial Measurements. J. Am. Chem. Soc..

[6] Velve-Casquillas G., Fu C., Le Berre M., Cramer J., Meance S., Plecis A., Baigl D., Greffet J.J., Chen Y., Piel M. (2011). Fast microfluidic temperature control for high resolution live cell imaging. Lab Chip.

[7] Velve-Casquillas G., Costa J., Carlier-Grynkorn F., Mayeux A., Tran P.T. (2010). A Fast Microfluidic Temperature Control Device for Studying Microtubule Dynamics in Fission Yeast. Methods Cell Biol..

[8] Scorzoni A., Tavernelli M., Placidi P., Zampolli S. (2015). Thermal Modeling and Characterization of a Thin-Film Heater on Glass Substrate for Lab-on-Chip Applications. IEEE Trans. Instrum. Meas..

[9] Moschou D., Vourdas N., Kokkoris G., Papadakis G., Parthenios J., Chatzandroulis S., Tserepi A. (2014). All-plastic, low-power, disposable, continuous-flow PCR chip with integrated microheaters for rapid DNA amplification. Sens. Actuators B Chem..

[10] Jiao Z., Huang X., Nguyen N.-T., Abgrall P. (2008). Thermocapillary actuation of droplet in a planar microchannel. Microfluid. Nanofluid..

[11] Wyzkiewicz I., Grabowska I., Chudy M., Brzozka Z., Jakubowska M., Wisniewski T., Dybko A. (2006). Self-regulating heater for microfluidic reactors. Sens. Actuators B Chem..

[12] Buser J.R., Diesburg S., Singleton J., Guelig D., Bishop J.D., Zentner C., Burton R., LaBarre P., Yager P., Weigl B.H. (2015). Precision chemical heating for diagnostic devices. Lab Chip.

[13] Weigl B., Domingo G., LaBarre P., Gerlach J. (2008). Towards non- and minimally instrumented, microfluidics-based diagnostic devices. Lab. Chip.

[14] Miralles V., Huerre A., Malloggi F., Jullien M.-C. (2013). A Review of Heating and Temperature Control in Microfluidic Systems: Techniques and Applications. Diagnostics.

[15] Craw P., Balachandran W. (2012). Isothermal nucleic acid amplification technologies for point-of-care diagnostics: A critical review. Lab Chip.

[16] Nunes P.S., Ohlsson P.D., Ordeig O., Kutter J.P. (2010). Cyclic olefin polymers: Emerging materials for lab-on-a-chip applications. Microfluid. Nanofluid..

[17] Mannella G.A., la Carrubba V., Brucato V. (2014). Peltier cells as temperature control elements: Experimental characterization and modeling. Appl. Therm. Eng..

[18] Wang Y., Song H., Pant K. (2014). A reduced-order model for whole-chip thermal analysis of microfluidic lab-on-a-chip systems. Microfluid. Nanofluid..

[19] Kumar S., Cartas-Ayala M.A., Thorsen T. (2013). Thermal modeling and design analysis of a continuous flow microfluidic chip. Int. J. Therm. Sci..

[20] Pardy T., Rang T., Tulp I. (2015). Finite element modelling of the resistive heating of disposable molecular diagnostics devices. Computational Methods and Experimental Measurements XVII.

[21] Pardy T., Rang T., Tulp I. Modelling and experimental characterisation of thermoelectric heating for molecular diagnostics devices. Proceedings of the 2016 15th Biennial Baltic Electronics Conference Electronics Conference (BEC).

[22] Pardy T., Tulp I., Rang T. Finite Element Modelling for the Optimization of Microheating in Disposable Molecular Diagnostics. Proceedings of the 14th International Conference on Simulation and Experiments in Heat Transfer and its Applications.

[23] Von Meier A. (2006). Electric Power Systems: A Conceptual Introduction.

[24] Kandlikar S., Garimella S., Li D., Colin S., King M.R., Kandlikar S.G. (2006). Heat Transfer and Fluid Flow in Minichannels and Microchannels.

[25] Griffiths D.J. (1999). Introduction to Electrodynamics.

[26] Rood P. (1928). A Visual Method of Showing The High Temperature Coefficient of Resistance of Metals as Compared with Alloys. J. Opt. Soc. Am..

[27] Steinhart J.S., Hart S.R. (1968). Calibration curves for thermistors. Deep Sea Res. Oceanogr. Abstr..

[28] Srivastava M., Srivastava M.C., Bhatnagar S. (2009). Control Systems.

[29] Friess H., Haussener S., Steinfeld A., Petrasch J. (2013). Tetrahedral mesh generation based on space indicator functions. Int. J. Numer. Methods Eng..

[30] DBK David+Baader GmbH (2009). HP Series of PTC Heaters. http://www.farnell.com/datasheets/11488.pdf.

[31] Shioi R., Umeya K., Yonezuka K., Senzaki H. (1981). Heating Element Made of PTC Ceramic Material. U.S. Patent.

[32] Huybrechts B., Ishizaki K., Takata M. (1995). The positive temperature coefficient of resistivity in barium titanate. J. Mater. Sci..

[33] Minco Products Inc (2007). Flexible Heaters Design Guide HDG01121806(A).

